# Molecular recognition. 1. Crystal structures of hexaazamacrocyclic amines containing *p*-xylylene spacers and their adducts with acids

**DOI:** 10.1186/1860-5397-1-16

**Published:** 2005-12-09

**Authors:** Teresa Borowiak, Grzegorz Dutkiewicz, Maciej Kubicki, Marek Pietraszkiewicz, Agnieszka Gil, Rainer Mattes

**Affiliations:** 1 Faculty of Chemistry, Adam Mickiewicz University, Grunwaldzka 6, 60-780 Poznań, Poland; 2 Institute of Physical Chemistry, Polish Academy of Sciences, Kasprzaka 44/52, 01-224 Warszawa, Poland; 3 Institute of Inorganic and Analytical Chemistry, University Muenster Corrensstrasse 36, 48149 Muenster, Germany

**Keywords:** Crystal structures, Macrocyclic amines, Fumaric adduct of, Hydrogen bonding, Molecular recognition, Two-dimensional supramolecular frameworks

## Abstract

The macrocyclic amine, 1,5,9,18,22,26-hexaaza [11.11]-*p*-cyclophane (**1**) contains two dipropylenetriamine units which make the molecule highly basic. Owing to this basicity, **1** can be highly protonated in acidic media and can form supramolecular assemblies with different anions. Two new supramolecular structures arise from self-assembly of the salt of **1** with fumaric acid and of the N-methyl derivative of **1**.

## Introduction

Macrocyclic polyamines are well-known to participate in molecular recognition phenomena with different kinds of substrates such as organic and inorganic anions or neutral molecules[1] that give rise to environmental, industrial or health-related potential applications.[2] Macrocyclic molecules containing two polyamine chains linked by different spacers are also capable to form complexes with metal ions in close proximity which is considered to be an important factor in increasing the efficiency in activation of substrates. This leads to numerous properties as magnetic, electronic or catalytic. [1,3–5]

**Scheme 1:**
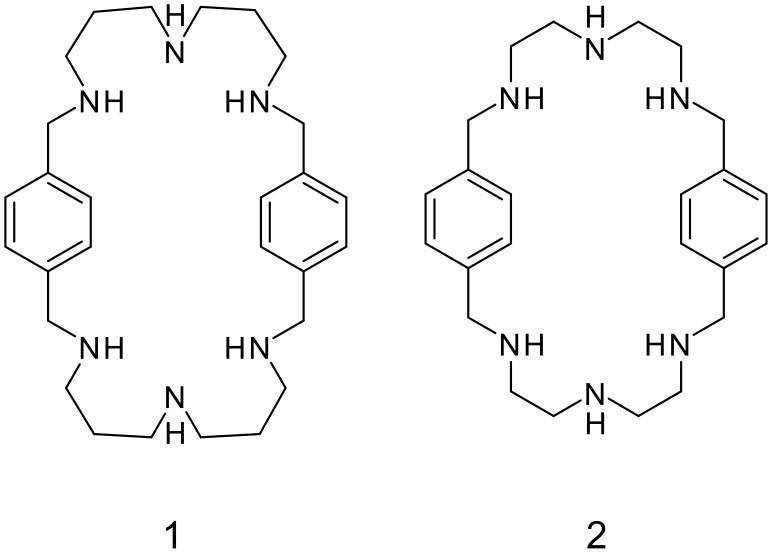
Formulas of macrocyclic amines, **1**: 1,5,9,18,22,26-hexaaza [11.11]-*p*-cyclophane and **2**: 1,4,7,16,19,22-hexaaza [9.9]-*p*-cyclophane.

In our previous paper we described the supramolecular structures of 1,5,9,18,22,26-hexaaza [11.11]-*p*-cyclophane (hereafter **1**, Scheme 1) complexes with *o*-nitrophenol and hydrochloric acid.[6] This symmetrical macrocyclic amine is built from two dipropylenetriamine units connected by two *p*-xylylene spacers and its all six nitrogens are strongly to medium-strongly basic.[2] The high basicity of this ligand is mainly determined by the number of methylene units bonded within each secondary amine with a lesser effect produced by the spacer. As the number of methylene units increases, the basicity of the macrocyclic ligands also increases because the amino-groups lie further apart from one another [2] in comparison with 1,4,7,16,19,22-hexaaza [9.9]-*p*-cyclophane (hereafter **2**, Scheme 1) which contains the shorter ethylene groups between its nitrogen atoms.

Supramolecular assemblies of **2** with anions were extensively studied by X-ray diffraction. [7–11] Due to the high basicity **1** can be highly protonated in acidic media and can also form supramolecular assemblies with different anions. However, structural information on the complexes of **1** with anions is scarce in the Cambridge Database.[12]

Only two types of protonation of **1** have been found so far. With the very weak acid – *o*-nitrophenol **1** forms a tetra-protonated salt whereas with hydrochloric or hydrobromic acids a hexaprotonated cation is produced.[6,13] The supramolecular assemblies which are formed between the receptor **1** and the anionic guests under participation of solvent molecules are kept together by two main types of interactions: Coulombic forces and hydrogen bonding. Host-guest interactions depend also strongly on the third factor – the geometrical fit of the anions to the receptor, whose conformation has to be adapted in order to maximize the mutual interactions. As the molecule **1** is highly flexible due to its long dipropylenetriamine units, its conformation can easily be matched with the all packing requirements. The structures of complexes of **1** with hydrochloric and hydrobromic acids as well as with *o*-nitrophenol show this ability: in the tetra-protonated complex the conformation adopted by the cation is a chair-like whereas the hexa-protonated cation adopts a rectangular conformation.[6,13]

The solvent molecules play a significant role in the supramolecular structure – they form its integral part, by filling up the empty space and by participating in hydrogen bonding interactions.

In the current paper the results of X-ray investigations of a complex of **1** with fumaric acid (*trans*-2-butenedioic acid, hereafter **1-FUM**) and of the N-methylated derivative of **1**, 1,5,9,18,22,26-hexamethyl-1,5,9,18,22,26-hexaaza [11.11]-*p*-cyclophane (hereafter **1-N-Me**) are presented.

## Results and Discussion

**Figure 1:**
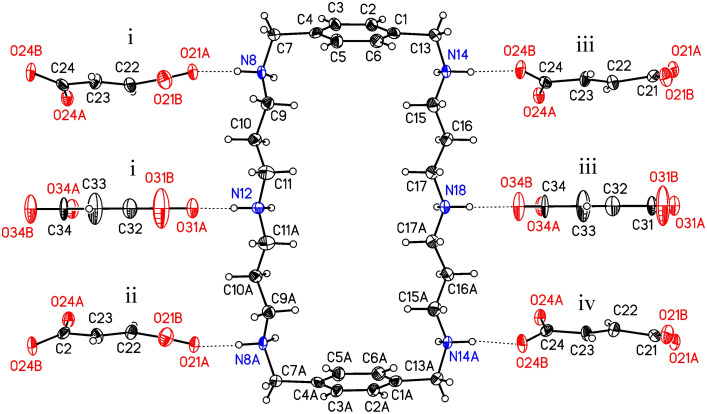
The ionic synthon of **1-FUM** built from one macrocyclic hexa-protonated cation and six fumaric anions, together with the atom labeling scheme. Displacement parameters are drawn at 50% probability level. Symmetry codes: i: 0.5-x, 0.5+y, z; ii: 0.5-x, 0.5+y, 0.5-z; iii 0.5-x, -0.5+y, z; iv: 0.5-x, -0.5+y, 0.5-z

The ionic components of **1-FUM** comprise a hexa-protonated cation (C_28_H_52_N_6_)^6+^ with its N(12)^+^ and N(18)^+^ lying across the plane of symmetry in the space group Pbnm, three fumaric anions, one of them occupying the same special position across the plane of symmetry and seven water molecules. The ionic synthon is shown in Figure 1.

The hexa-protonated cation adopts a rectangular conformation similar to that in the structure of **1** with hydrochloric or hydrobromic acids,[6,13] with all twelve N-H bonds directed out of the macrocycle. Six of them are roughly normal to the macrocyclic ring whereas the other six are located approximately in the average plane of the macrocycle and hence the cation acts as a twelve-fold donor in hydrogen bond formation. Looking at the dihedral angles in the aliphatic part of the cation, one finds two gauche and eight anti-bonds in each chain. The mutual atomic arrangement in both chains is close to *C*_2v_ (mm2) point symmetry, with the additional non-crystallographic plane of symmetry perpendicular to the crystallographic one. The height of the macrocyclic cation – the distance between the phenyl ring centroids equals 12.61(1) Å and the width – the N(12)^+^ – N(18)^+^ distance of 6.84(1) Å, are similar to those found for the salt with hydrochloric acid. Due to the crystallographic plane of symmetry no shift between the centroids of the phenyl rings exists, they are oriented in an antiparallel mode with the angle between their planes of 43.1(1)°.

The observed C-N bond lengths range from 1.483(5) to 1.506(5) Å and confirm protonation of all N atoms. Moreover, all the hydrogen atoms at nitrogens were found in the Δ*F* maps.

The fumaric anion in general position is not planar whereas the anion in the special position displays a statistically averaged planarity with high displacement parameters, as a result of dynamic disorder.

**Figure 2:**
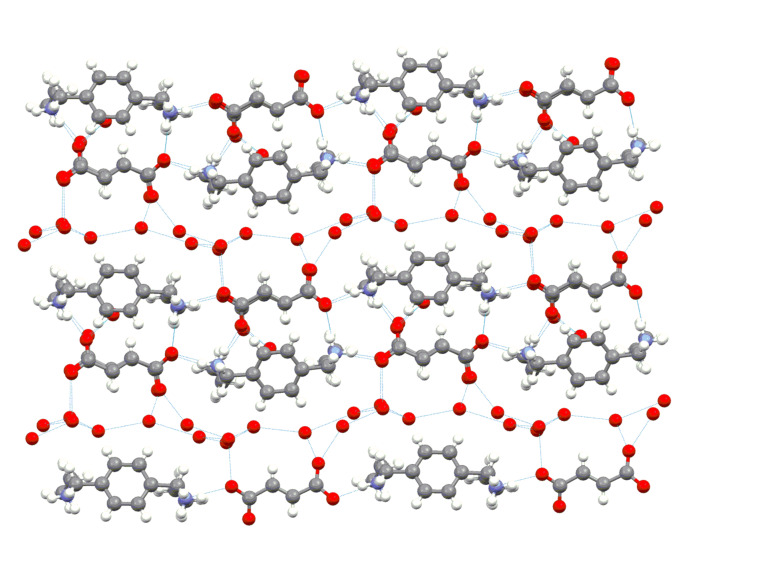
The structure of one sheet in **1-FUM** – a projection perpendicular to the phenyl ring planes. Tapes of water molecules separate tapes built from macrocyclic cations and fumaric anions. Hydrogens of water molecules haven't been located (N-atoms in blue, O atoms in red).

The supramolecular structure of **1-FUM** is unique and different from those which were found in crystals of **1** with HCl, HBr and *o*-nitrophenol. It clearly shows that the macrocycle adjusts its conformation in order to optimize Coulombic interactions with the dianion of fumaric acid. Thus, as in the previous examples,[6,13] the supramolecular structure is organized due to the shape of the macrocyclic component into two-dimensional sheets whose heights are the same as the height of the macrocyclic cation, i.e. 12.61 Å and no more similarity exists between the supramolecular structures of **1-FUM** and of **1** with HCl, HBr, and *o*-nitrophenol. Within the sheet, intermolecular hydrogen bonds are ubiquitous (Table 1) and arrange the ionic components into tapes with a zigzag pattern which results from the geometrical fit. The structure of one sheet is shown in Figure 2.

**Table 1:** Hydrogen bonds for **1-FUM** [Å and °].

D-H···A	d(D-H)	d(H···A)	D(D···A)	<(DHA)

N8-H8A···O21A^i^	0.90	1.83	2.718(5)	167
N8-H8B···O24A	0.90	1.88	2.754(6)	164
N12-H12A···O31A^i^	0.90	1.76	2.640(8)	166
N12-H12B···O34A	0.90	1.87	2.760(8)	167
N14-H14A···O21A	0.90	1.89	2.770(6)	167
N14-H14B···O24B^ii^	0.90	1.85	2.713(5)	161
N18-H18A···O31A	0.90	1.84	2.734(8)	170
N18-H18B···O34B^ii^	0.90	1.82	2.699(8)	166

Symmetry transformations used to generate equivalent atoms: ^i^ -x+1/2, y+1/2, z; ^ii^ -x+1/2, y-1/2, z

**Figure 3:**
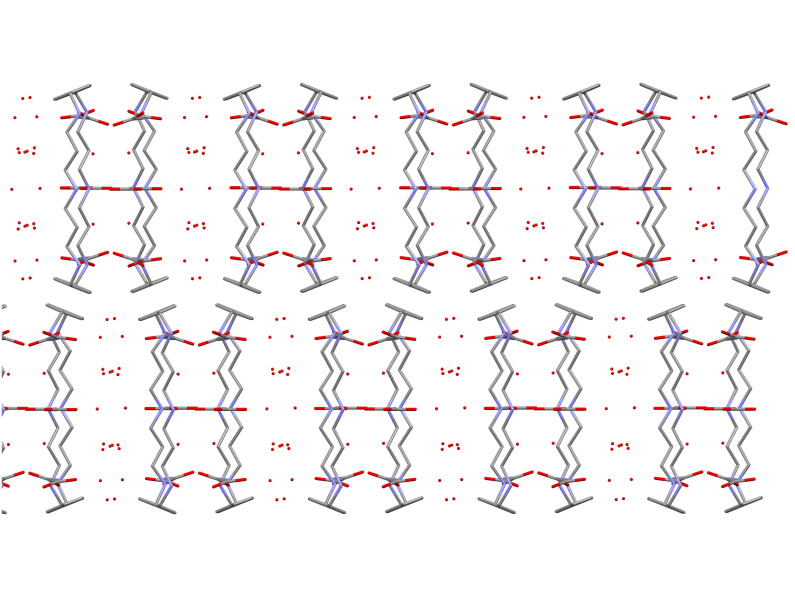
The arrangement of two sheets (after rotation by 90°). The height of one sheet corresponds to the height of the macrocycle, i.e. about 12 Å.

Thus, one of the carboxylic groups of the anion forms a bifurcated hydrogen bonding N^+^- H···O^-^ to two N^+^-H groups which come from two different macrocycles. Hereby the zigzag arrangement mentioned above is formed. A second bifurcated hydrogen bonding is formed to two different water molecules which fill up the empty space between two neighbouring tapes (Figure 2). Further, each of two C-O^-^ bonds of the second carboxylic group are hydrogen bonded by bifurcated bonds to another N^+^- H group and a water molecule (Figure 2). Water molecules located in channels form also infinite tapes by intermolecular hydrogen bonding. Their hydrogen atoms were not found in the Δ*F* maps probably due to the disorder. The infinite water channels have the same height as the sheet height and no interactions exist between water channels belonging to different sheets (Figure 2 and Figure 3). It's worth mentioning that no hydrogen bonds are formed between water molecules and cations.

**Figure 4:**
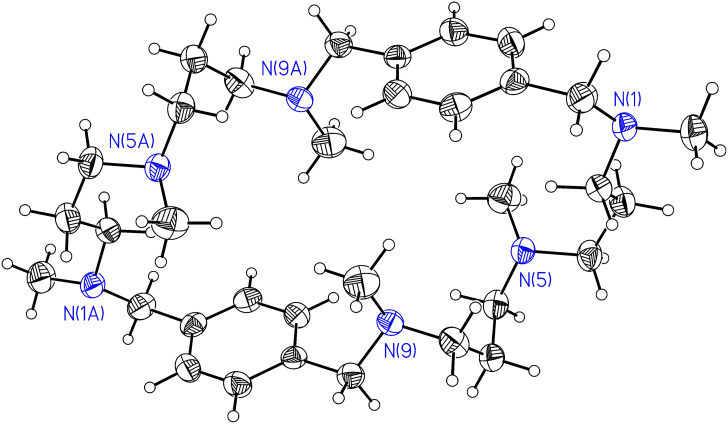
The molecular conformation of **1-N-Me**, atom labeling scheme indicated. Displacement parameters are drawn at 50% probability level.

The crystal structure of **1-N-Me** contains isolated centrosymmetric molecules. The molecular conformation together with the atom labeling scheme is shown in Figure 4.

The overall conformation of the molecule is ellipsoidal with the approximate dimensions of 6.2 and 15.3 Å. All atoms of the molecule (except hydrogens) are within 2.16 Å of the mean molecular plane. Four of the six methyl groups are directed towards the molecular cavity. They are situated in pairs slightly above and below the cavity due to the crystallographic symmetry. The remaining two methyl groups are exo-oriented along the long molecular axis.

No π-π interactions occur in the structure, neither inter- nor intramolecular. All interatomic distances and bond angles scatter only slightly and are quite normal, e.g. the C-N distances range from 1.456(2) – 1.465(2) Å. A slight deviation from tetrahedral bonding is observed at the nitrogen atoms. The mean value of the angle between the bonds originating at these atoms is expanded to 111.4(1)°. The torsion angles within each of the aliphatic parts of the molecule show six gauche and four anti-oriented bonds, the latter mainly at the nitrogen atoms.

**Figure 5:**
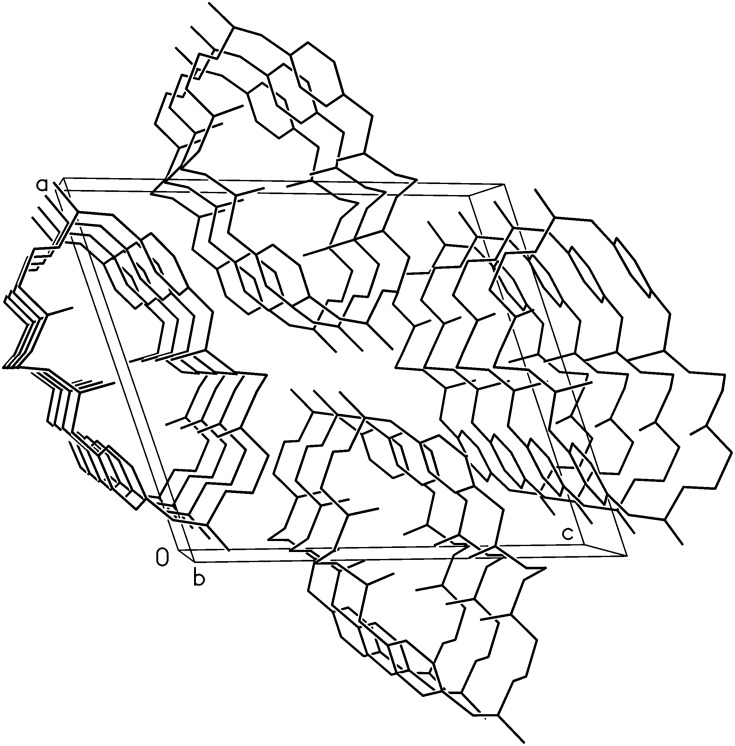
The unit cell of **1-N-Me**, stacks of molecules are indicated.

The rather flat molecules are stacked in the lattice along the b-axis (Figure 5). The supramolecular structure is similar to that of **2**[8] although in the last one water molecules co-crystallize with the chair-like molecules of **2**.

## Description of Supporting Information

Crystallographic data for the structural analysis have been deposited with the Cambridge Crystallographic Data Centre, CCDC 279700 for compound **1-FUM** and CCDC 203505 for compound **1-N-Me**. Copies of the information may be obtained free of charge from the Director, CCDC, 12 Union Road, Cambridge CB2 1EZ, UK (Fax: +44-12323-336-033; E-mail: deposit@ccdc.cam.ac.uk or www: http://www.ccdc.cam.ac.uk).

## Supporting Information

File 1 Experimental and crystallographic data together with data collection and structure refinement details.
