# Supplementary material for: Molecular recognition. 1. Crystal structures of hexaazamacrocyclic amines containing p-xylylene spacers and their adducts with acids
Source: Beilstein J Org Chem. 2005 Dec 9;1:16. doi: 10.1186/1860-5397-1-16 (PMC1399464; doi:10.1186/1860-5397-1-16)
Supplement: File 1 — Experimental and crystallographic data together with data collection and structure refinement details. [file Beilstein_J_Org_Chem-01-16-s001.doc]

# Experimental

*Synthesis*

The macrocyclic amine **1** and its N-methylated derivative **1-N-Me** were obtained in high yield according to the procedure described by Pietraszkiewicz and Gąsiorowski ^1^. The analytical and spectral characteristics of both compounds were also described by Pietraszkiewicz and Gąsiorowski^1^. The adduct of **1** with fumaric acid (**1-FUM**) was obtained in the following way: one equivalent of the amine **1** and three equivalents of fumaric acid were dissolved in water and then ethanol was added until a small amount of precipitate was obtained. Next, the mixture was warmed up until the precipitate was dissolved. The reaction mixture was cooled down slowly and after two days colourless prismatic crystals were obtained.

The hexamethyl derivative of **1** (**1-N-Me**) was crystallized from acetonitrile to obtain colourless prismatic crystals.

*Data collection, structure solution and refinement of* **1-FUM**.

A colorless prism of dimensions 0.1 x 0.15 x 0.45 mm was chosen for X-ray diffraction experiment. Diffraction data were collected on a KUMA KM4CCD diffractometer using graphite monochromated MoK_α_ radiation at 150(2) K. Low temperature was controlled with Oxford Cryosystem cooling device. The measurements were done in six separate runs in order to cover symmetry independent part of the reciprocal space. The ω-scan was used with the step of 0.75^o^, two reference frames were measured after every 50 frames, they did not show any systematical changes either in the peak positions or in their intensities. Total of 782 frames were collected. The unit cell parameters were determined by least-squares treatment of the setting angles of 1880 highest intensity reflections, chosen from the whole experiment. The Lorentz and polarization corrections were applied ^2^. The structure was solved by direct methods with SHELXS-97 program ^3^ and refined by full matrix least-squares on *F^2^* , using SHELXL-97 program ^4^. Non-hydrogen atoms were refined anisotropically. Positions of all hydrogen atoms were calculated in their idealized positions and refined using a riding model. Isotropic displacement parameters were fixed at 1.2 of U_eq_ of their parent non-hydrogen atoms. Scattering factors incorporated in SHELXL-97 were used. The function Σ*w*( *F_o_* ^2^ -  *F_c_* ^2^ )^2^ was minimized with *w*^-1^ = [σ^2^(*F_o_*)^2^ + (0.17*P*)^2^]. Molecular graphics was performed with standard programs ^5^. Relevant crystallographic data together with data collection and structure refinement details are listed in Table 1.

*Data collection, structure solution and refinement of* **1-N-Me**

A colourless prismatic crystal with the dimensions 0.1 x 0.15 x 0.3 mm was chosen for X-ray diffraction experiment. Data were collected on an Enraf Nonius Kappa CCD area detector at 198(2) K using graphite monochromated MoK_α_ radiation.

A total of 10747 reflection data were collected, which gave 3974 independent data ((R_int_ = 0.0454) after a SORTAV absorption correction (absorption coefficient = 0.065 mm^-1^, T_min_ = 0.9808, T_max_ = 0.9936). The structure was solved by direct methods with SHELXS-97 program ^3^ and refined by full matrix least-squares on *F^2^*, using SHELXL-97 program ^4^. A total of 184 parameters were refined. Hydrogen atoms were treated according the riding model. The final R-indices are R_1_ = 0.0558 and wR_2_ = 0.1151 for reflections with I>2sigma(I). The crystallographic data together with data collection and structure refinement details are listed in Table 1.

**References**

1. Pietraszkiewicz M, Gąsiorowski R: *Chem. Ber.* 1990, **123**: 405-406.
2. *CrysAlis CCD*, Oxford Diffraction , 2002.
3. Sheldrick GM: *Acta Crystallogr.* 1990, A**46**: 467-473.
4. Sheldrick GM: *SHELXL-97*, University of Gőttingen 1997.
5. Siemens, Stereochemical Workstation. Siemens Analytical X-ray Instruments Inc., Madison, Wisconsin, USA, 1989.

**Table 1. Crystal data and structure refinement for 1-FUM and** 1-N-Me

| Identification code | **1-FUM** | **1-N-Me** |
| --- | --- | --- |
| Empirical formula | [C_28_H_52_N_6_]^6+^ · 3[C_2_H_2_(COO)_2_]^2-^ · 7H_2_O | C34 H58 N6 |
| Formula weight | 1022.92 | 550.86 |
| Temperature | 150(2) K | 198(2) K |
| Wavelength | 0.71073 Å | |
| Crystal system | Orthorhombic | Monoclinic |
| Space group | Pbnm | P2_1_/n |
| Unit cell dimensions | a = 11.028(1) Å | a = 15.975(1) Å |
|  | b = 17.714(2) Å | b = 6.277(1) Å, β = 108.84(1)° |
|  | c = 28.650(3) Å | c = 17.744(1) Å |
| Volume | 5596.8(10) Å^3^ | 1684.0(3) Å^3^ |
| Z | 4 | 2 |
| Density (calculated) | 1.214 Mg/m^3^ | 1.086 Mg/m^3^ |
| Absorption coefficient | 0.102 mm^-1^ | 0.065 mm^-1^ |
| F(000) | 2160 | 608 |
| Crystal size | 0.1 x 0.15 x 0.45 mm | 0.30 x 0.15 x 0.10 mm |
| Theta range for data collection | 5.01 to 22.5°. | 1.49 to 27.87°. |
| Index ranges | -15≤h≤15, -23≤k≤19, -37≤l≤38 | -15≤h≤21, -8≤k≤7, -23≤l≤23 |
| Reflections collected | 32912 | 10474 |
| Independent reflections | 3687 [R(int) = 0.0585] | 3974 [R(int) = 0.0454] |
| Completeness | to Θ = 22.5°- 98.5 % | to Θ = 27.87° - 99.0 % |
| Refinement method | Full-matrix least-squares on F^2^ | |
| Data / restraints / parameters | 3687 / 0 / 337 | 3974 / 0 / 184 |
| Goodness-of-fit on F^2^ | 1.145 | 1.023 |
| Final R indices [I>2sigma(I)] | R1 = 0.0869, wR2 = 0. 2567 | R1 = 0.0558, wR2 = 0.1151 |
| R indices (all data) | R1 = 0. 1333, wR2 = 0.2864 | R1 = 0.1133, wR2 = 0.1371 |
| Largest diff. peak and hole | 1.290and -0.599e·Å^-3^ | 0.159 and -0.213 e·Å^-3^ |
